# Biomarkers, diagnosis, and the meaning of disease: evaluating competing frameworks for Alzheimer's disease classification

**DOI:** 10.3389/fnagi.2026.1841695

**Published:** 2026-06-18

**Authors:** Ethan Terman

**Affiliations:** Graduate School of Arts and Sciences, New York University, New York, NY, United States

**Keywords:** Alzheimer's disease, asymptomatic labeling, biomarkers, diagnostic frameworks, international working group, NIA-AA, preclinical AD

Few questions in contemporary neurology carry more consequence than how Alzheimer's disease (AD) should be defined. For much of the 20th century, AD was understood as a clinical syndrome: dementia of insidious onset, progressive course, and characteristic neuropsychological features. The clinician's task was to recognize the syndrome, exclude competing etiologies, and support the patient. Diagnosis and disease were coextensive with observable impairment. That understanding is now being fundamentally revised. Advances in amyloid and tau biomarker science have made it possible to detect neuropathologic changes years, and potentially decades, before cognitive symptoms emerge, creating pressure to reconceptualize AD as a biological entity definable in the absence of clinical illness. The resulting tension between two major international frameworks, the 2024 revised criteria of the National Institute on Aging and Alzheimer's Association (NIA-AA) and the contemporaneous recommendations of the International Working Group (IWG), reflects not merely a technical disagreement about diagnostic thresholds but a fundamental debate about what it means to have a disease.

The NIA-AA 2024 criteria define AD biologically, classifying individuals as having AD on the basis of abnormal core biomarkers, including plasma phosphorylated tau 217, cerebrospinal fluid amyloid beta 42/40 ratios, or amyloid positron emission tomography, regardless of whether cognitive symptoms are present (Jack et al., 2024). The framework organizes biomarkers into Core 1 markers, which establish biological diagnosis, and Core 2 markers, which refine staging and prognostic confidence. Symptom status modifies staging but does not define disease. This approach represents a deliberate alignment with fields such as oncology and cardiology, where presymptomatic biological abnormalities routinely justify diagnostic labels and preventive intervention. Its proponents argue that earlier identification will accelerate the development and deployment of disease-modifying therapies, enable informed life planning, and ultimately reduce dementia burden at a population level. These are not trivial considerations. The biological precision afforded by modern biomarkers represents a genuine scientific advance, and the aspiration toward presymptomatic intervention is clinically reasonable in principle.

The IWG framework, by contrast, maintains that AD is a clinical-biological construct requiring both biomarker evidence of AD pathology and clinically relevant cognitive impairment attributable to that pathology (Dubois et al., 2024). Cognitively unimpaired individuals with abnormal biomarkers are classified as asymptomatic at risk rather than as having the disease. Presymptomatic AD, in IWG terminology, is reserved for carriers of deterministic autosomal dominant mutations, where progression to clinical illness is effectively certain. This distinction is not semantic. It reflects a principled position that biological vulnerability and clinical illness are not equivalent, that the presence of amyloid plaques or tau tangles in an asymptomatic individual does not establish that disease, in any clinically meaningful sense, has occurred. The IWG framework resists collapsing this distinction in the absence of evidence that doing so reliably benefits patients.

The evidentiary basis for the NIA-AA position deserves careful scrutiny. Amyloid abnormality is present in approximately 24% of cognitively unimpaired older adults and in 51% to 61% of individuals with mild cognitive impairment, compared with 79% to 87% of those with clinical AD dementia (Jansen et al., 2022). These prevalence figures indicate that amyloid positivity is neither sufficient nor necessary for symptomatic disease. Longitudinal data further indicate that progression from isolated amyloid positivity to dementia is neither uniform nor rapid: many biomarker-positive individuals remain cognitively stable over clinically meaningful time horizons, and the modifiers of progression, including age, APOE genotype, cognitive reserve, and vascular comorbidity, remain incompletely understood. When biomarker-based diagnosis is extended to asymptomatic individuals, the probability that a given positive result reflects a trajectory leading to dementia in that individual's lifetime is substantially less than the aggregate prevalence statistics suggest. For practicing clinicians, translating a probabilistic population-level signal into guidance for an individual patient who presents without symptoms is an exercise in irreducible uncertainty.

These uncertainties carry direct clinical consequences. Diagnostic labels generate downstream effects that extend well beyond the clinical encounter. An AD diagnosis, even in the absence of symptoms, may affect psychological wellbeing and personal identity, influence employment and financial decisions, and carry implications for insurance eligibility and coverage (Karlawish, 2011). Autopsy-confirmed studies have demonstrated that a substantial proportion of patients diagnosed clinically with AD during life do not have AD pathology at death, a finding that should temper confidence in biomarker-based diagnosis in community settings where confirmatory testing may be unavailable or inconsistently applied (Beach et al., 2012). As anti-amyloid therapies enter broader clinical use, the stakes of diagnostic expansion will increase further. Lecanemab and donanemab have demonstrated statistically significant but modest slowing of decline in early symptomatic populations, and their risk profiles, including amyloid-related imaging abnormalities characterized by edema or microhemorrhage, are not trivial. Critically, evidence from presymptomatic intervention trials remains limited and inconclusive. The A4 Study, which enrolled cognitively unimpaired amyloid-positive adults and treated them with solanezumab, failed to demonstrate benefit on its primary cognitive endpoint, illustrating that biomarker positivity alone does not reliably identify individuals who will respond to anti-amyloid intervention (Sperling et al., 2023). The ongoing A45 (AHEAD 3-45) trial with lecanemab in preclinical AD has not yet reported primary outcomes. Exposing asymptomatic individuals to these therapies on the basis of biomarker positivity alone, without clear evidence of net benefit in presymptomatic stages, introduces a meaningful potential for harm that current evidence cannot offset.

Biomarker discordance poses an additional practical challenge. Discrepancy rates between plasma biomarkers and cerebrospinal fluid or amyloid PET range from 10 to 31% depending on the cohort and modality, with higher discordance observed in prodromal stages and in populations with greater racial and ethnic diversity. In non-White populations, false-positive rates for plasma biomarkers have been reported as high as 42%, a figure derived from cross-sectional analyses comparing plasma phosphorylated tau 217 against PET confirmation in community-based cohorts with higher vascular comorbidity burdens; this estimate reflects a specific testing context and should be interpreted as an upper bound rather than a universal rate, though it highlights the degree to which validation cohort characteristics can substantially affect apparent diagnostic performance (Ashton et al., 2024). These disparities are not incidental. If a biological definition of AD is to guide clinical practice, its application must demonstrate validity across the populations it will be used to diagnose. The current evidence base is heavily weighted toward selected research cohorts with limited demographic representation, and extrapolation to broader populations risks overstating diagnostic certainty while systematically disadvantaging those already underserved by the healthcare system.

Neither framework is without limitation. The IWG criteria's requirement for clinical impairment may delay diagnosis in patients who would benefit from early intervention and may complicate recruitment for prevention trials that depend on identifying presymptomatic individuals. This concern is not merely theoretical: evidence from large multicenter cohorts demonstrates that individuals with significant amyloid burden but preserved cognition may represent a population with a narrowing therapeutic window, and that waiting for clinical impairment to emerge forecloses the opportunity to intervene before irreversible neurodegeneration occurs (Villemagne et al., 2013). Moreover, IWG criteria do not offer standardized guidance on the communication of asymptomatic at-risk status to patients, leaving clinicians without a clear framework for counseling biomarker-positive individuals who are not diagnosed with disease but who nonetheless carry prognostic uncertainty that may affect their psychological and social functioning (Karlawish, 2011). The NIA-AA framework's biological primacy, conversely, risks medicalizing uncertainty at scale and may outpace the clinical infrastructure needed to interpret and act on biomarker results responsibly. What both frameworks share is a recognition that AD biomarkers are clinically important and that their integration into diagnostic practice is both inevitable and necessary. The disagreement concerns how much weight those biomarkers should carry when clinical impairment is absent.

A resolution to this tension is unlikely to emerge from biomarker science alone. It will require prospective, population-based evidence demonstrating that a biological AD diagnosis in asymptomatic individuals reliably predicts clinically meaningful outcomes, that interventions initiated at presymptomatic stages confer net benefit, and that the downstream consequences of diagnostic labeling do not systematically harm those who are labeled. Until that evidence exists, the IWG framework's insistence on clinical-biological concordance as a prerequisite for diagnosis reflects an appropriate standard of epistemic caution. Biomarkers can and should inform prognosis, guide surveillance, and support clinical reasoning. They are not, by themselves, sufficient to define disease in individuals who remain cognitively and functionally intact. In the interim, several operational steps can help navigate the gap between biomarker science and clinical readiness. Clinicians should document asymptomatic biomarker positivity using established risk communication frameworks, explicitly distinguishing probabilistic risk from disease diagnosis and providing structured follow-up pathways with defined reassessment intervals. Specialty memory clinics and primary care networks should develop shared protocols for biomarker counseling that address psychological impact, insurance implications, and appropriate referral for preventive lifestyle interventions with evidence of cognitive benefit (Karlawish, 2011). Enrollment in prevention trials should be actively facilitated for biomarker-positive individuals without symptoms, as this represents the most responsible pathway for generating the evidence that both frameworks acknowledge is needed. Regulatory agencies and professional societies should establish minimum reporting standards for biomarker test performance across demographic subgroups before results are used to guide treatment decisions in non-research settings. The history of medicine offers repeated cautionary examples of diagnostic expansion outpacing the evidence for benefit. Alzheimer's disease, given the scale of the populations potentially affected and the severity of the interventions now available, is not a domain in which that lesson can be safely ignored (Sperling et al., 2011).
